# Integrating complementary and alternative medicine in surgical care: A narrative review

**DOI:** 10.1097/MD.0000000000040117

**Published:** 2024-10-11

**Authors:** Nasser Hakami

**Affiliations:** a Surgical Department, College of Medicine, Jazan University, Jazan, Saudi Arabia.

**Keywords:** acupuncture, complementary and integrative medicine, postoperative care, preoperative care, rehabilitation, surgical care

## Abstract

Complementary and integrative medicine (CIM) is increasingly being integrated into preoperative, intraoperative, and postoperative phases to enhance patient outcomes, manage symptoms, and improve overall well-being. CIM encompasses a broad range of therapies and practices that are not typically part of conventional medical care, such as herbal and non-herbal medicine, yoga, acupuncture, meditation, chiropractic care, and dietary supplements. This review explores the existing evidence on the application, benefits, and challenges of CIM therapies and practices in surgical settings, highlighting the importance of integrating these therapies and approaches with conventional medical practices to enhance patient outcomes.

## 1. Introduction

Complementary and integrative medicine (CIM) encompasses a variety of therapies and practices that are utilized alongside conventional medical therapies to care for the whole person.^[1]^ Numerous studies have established that the public use of CIM has grown dramatically since the 1980s in industrial countries.^[2–5]^ Several definitions for CIM have been proposed, but no one has reached a consensus.^[6]^ The terms alternative, integrative, and complementary are continually evolving. The National Center for Complementary and Integrative Medicine, defines complementary medicine as: “a group of diverse medical and health care systems practices, and products that are not generally considered part of conventional medicine.”^[1,7]^ National Center for Complementary and Integrative Medicine further referred to the distinction between Integrative and Alternative medicine as “Integrative” refers to use of complementary together with conventional medicine (CM), such as using acupuncture in addition to usual care to help lessen pain, while “Alternative” refers to the use of complementary in place of CM. In a systematic review of 16 papers, the average prevalence of CIM was 32.2%.^[4]^ In the meantime, it was estimated that 62.5% of type 2 diabetes mellitus patients in primary care settings and 70.2% of cancer patients in Malaysia used CIM.^[8,9]^ CIM uses reported more in cancer populations compared to other condition-specific populations.^[10–12]^

Based on the previous studies, the prevalence of CIM usage among surgery patients ranges between 3% and 90%, with a mean prevalence of 53%.^[13–18]^ CIM usage in general surgery are more often among women, younger group, and higher educated.^[19–23]^ However, Lucenteforte in Italy reported that older surgery patients used herbal remedies to a greater extent in comparison to the younger group.^[18]^ Schiff et al proposed strategies for integrating CIM care within the surgery for hospitalized patients, inspiring others who wish similarly to integrate CIM in their hospital services.^[24]^ As patients themselves use these therapies, there is a crucial need to investigate the effectiveness of such interventions in surgical care. This study examines the applicability, advantages, and difficulties of CIM therapies in surgical settings. The rationale behind reviewing the integration of a holistic health approach, combining conventional medical practices with complementary practices lies in improving patient outcomes, ensuring safety, fostering patient-centered care, and addressing the broader aspects of pain management, stress reduction, and overall well-being.

## 2. Methods

A comprehensive search was conducted across multiple electronic databases, including PubMed, MEDLINE, Cochrane Library, and Google Scholar. Keywords such as “Complementary and Integrative Medicine,” “CIM,” “surgical care,” “preoperative care,” “postoperative care,” “pain management,” and “rehabilitation” were used. Boolean operators were applied to refine the search (e.g., “CIM AND surgical care”). Studies that were randomized controlled trials (RCTs), cohort studies, case–control studies, systematic reviews, and meta-analyses, published in peer-reviewed journals, focused on the use of CIM in surgical settings, including preoperative, intraoperative, and postoperative phases and available in English, were included in the review. In this review, approximately 169 articles from peer-reviewed journals were selected based on their relevance to the integration of CIM in surgical care. No formal patient data aggregation was performed, as the focus was on synthesizing the broader trends and perspectives in the literature. Key topics emerging from the review include the clinical efficacy of CIM, patient satisfaction, and potential barriers to its adoption in surgical settings. Articles lacked a clear focus on surgical care, were non-peer-reviewed, with inadequate reporting or low methodological quality were excluded. The methodological quality of the studies was assessed using the Cochrane Risk of Bias Tool for randomized trials and the Newcastle-Ottawa Scale for cohort and case–control studies. A narrative synthesis was employed to summarize findings across studies.

## 3. Discussions/observations

### 3.1. Patient and healthcare providers (HCP) perspectives

Several studies reveal the lack of communication between the patients and HCP related to CIM uses and in fact patient usually do not stigmatize their CIM usage.^[19,21]^ In some studies, patients expressed their interest in integrating of CIM during their surgical care.^[14,22]^

There are many reasons behind the extensive use of CIM among patients as reported in many previous controlled trials and systematic reviews.^[25,26]^ The perceived benefits and safety of CIM, socio-culture alignment and beliefs and needs are the main reasons for CIM use among 5 systematic reviews.^[10,27–30]^ Influence from relatives, friends, and the social network, accessibility and availability of CIM, dissatisfaction with CM and perceived safety and benefits were reported in many other systematic reviews.^[31–34]^ According to a recent systematic review with many articles from various patient populations, some patients seek complementary and alternative medicine when their conditions cannot be adequately treated by CM or when CM has significant adverse effects.^[35]^ The perception and views of patients toward CIM are vital; however, HCPs beliefs, attitudes, and perspectives equally important for providing accurate risks and benefits, increasing patient involvement in decision-making, and refining treatment plans for better patient outcomes. Many previous studies have shown that, HCPs have low expectations and negative attitudes toward the use of CIM due to the concern of serious adverse effects and harmful interactions with conventional treatments.^[35–39]^ Conversely, similar studies note potential positive effects when using CIM.^[36,40–42]^ Nurses supported the use of complementary and alternative medicine more than any other professions for both its beneficial psychological benefits and its ability to improve quality of life.^[36,40,43]^ Research to assess perceptions of HCPs toward CIM usage in surgery is rare. Physicians, in one study in Germany,^[44]^ and nurses, in an American study^[45]^ feel that education in CIM is un necessary and they perceived it to be less important in acute surgical setting. In addition, one Australian study among nurses, demonstrates that half of the participants had very low awareness of CIM.^[46]^ In contrast, Brown et al in Canadian study reported that professionals perceived CIM to have an important role in pre- and postoperative setting.^[47]^ Surgical professionals in oncology setting, especially those who were also involved in research activity, are more likely to have positive perception toward CIM usage, in comparison to other specialties.^[48]^ Interestingly, females physicians revealed to be more positive towards CIM in obstetrics and gynecology, compared to male physicians.^[49]^ Shorofi, in an Australian study, reported that 50% of nurses in surgical ward practice CIM with hospitalized patients and the commonly practiced therapies were non-herbal supplements, aromatherapy, music therapy, massage, relaxation, and meditation techniques.^[19]^ CIM practices, including acupuncture, music therapy, hypnosis, and aromatherapy, have been shown to be effective at reducing preoperative anxiety, analgesic consumption, postoperative nausea and vomiting, and postoperative pain, for patients undergoing otolaryngologic procedures.^[50]^

### 3.2. Types of CIM therapies

Complementary therapies can be broadly grouped according to their primary therapeutic focus.^[1]^ These include nutritional approaches, such as specialized diets, supplements, herbal remedies, and probiotics; psychological methods, such as mindfulness practices; and physical therapies, such as massage or spinal manipulation. Some approaches combine elements from different domains, like yoga, tai chi, and acupuncture, which incorporate both physical and psychological components, or mindful eating, which merges psychological and nutritional strategies. Additionally, expressive therapies like dance or art therapy blend psychological and physical dimensions. CIM therapies such as acupuncture, yoga, massage, hypnosis, and music therapy, as well as prayer, and herbal and non-herbal medicine, have previously reported in surgical care.^[51–53]^

#### 3.2.1. Nutritional approaches

These approaches involve a range of products, such as vitamins and minerals, herbs, and probiotics.^[54]^ Herbal medicine or herbal supplements, including Black cohosh, Echinacea, Evening primrose, Feverfew, and Garlic, are plant-based products used to treat or prevent disease and enhance general health and wellbeing (Table 1).^[55]^ The majority of them come from traditional Chinese herbal medicine and has been reviewed extensively in the literature.^[56,78]^ Previous reviews suggest significant benefits and promising effects in surgical practice.^[57–59,78]^ An increasing number of Chinese herbal supplements have been reported to have significant benefits in treatment of acute pancreatitis.^[57,58]^ Clinical studies proved that Chinese herbal medicines (CHMs) could decrease pro-inflammatory cytokines^[60]^ and through eliminating excessive reactive oxygen species, relieving the necrosis in acinar cells and inducing apoptosis, they could also render pancreas more resilient to stress and microcirculation disorder.^[61]^ In addition, CHMs could promote the gastrointestinal motility and the recovery of intestinal mucosal permeability, and hence decrease the incidence of acute pancreatitis.^[60]^

**Table 1:** Herbal supplements used in surgical care.

Type of nutrient	Surgical setting/use	References
Purple coneflower (*Echinacea purpurea*)	Boosts immune function, potentially reducing infection risk post-surgery	[13,55–66]
Garlic (*Allium sativum*)	Promotes heart health and reduces infection risk	[13,55–62,64–66]
St. John’s wort (*Hypericum perforatum*)	Enhances inflammatory response and used for better healing	[13,55–62,67–70]
Maidenhair tree (*Ginko biloba*)	Anti-inflammatory, antioxidant, and neuroprotective properties	[13,56–62,64–66,68,71]
Ginseng (*Panax ginseng*)	Anti-inflammatory effects, potentially improving functional recovery after surgery	[13,56–62,67,68,72]
Glucosamine (C_6_H_13_NO_5_)	Enhances recovery, supports gut function	[13,56–62,64–66,73]
Evening primerose oil (*Oenathera biennis*)	Safe potential adjunctive treatment for postoperative pain control	[13,56–62,74,75]
Aloe vera	Prevention and healing of skin wounds and proved to be effective in the management of postoperative complications, especially pain and swelling	[13,56–62,76,77]
Saw Palmetto (*Serenoa repens*)	Used to treat urinary and reproductive system problems and Benign prostatic hyperplasia (BPH)	[13,56–62,64]
Fish/cod liver oil	Have anti-inflammatory properties helped in reducing postoperative complications	[14,65–68]

For patients with functional dyspepsia, meta-analyses indicated that CHMs were more effective than prokinetic agents for the alleviation of global dyspeptic symptoms and considered to be an alternative to alleviation of functional dyspeptic symptoms when proton pump inhibitors and prokinetic agents are contraindicated.^[62,79–81]^ Evidence revealed that CHMs performed slightly better in reducing fullness sensation, reducing epigastric pain and burning and could enhance better gastric emptying relieve and relieve early satiety. On the other hands, many medicinal plants, including peppermint and caraway are increasingly used in the management of non-ulcer dyspepsia.^[82,83]^

Extracts from the plants used in the management of functional constipation,^[84]^ exhibit laxative effects through the release of nondigestible polysaccharides as in *Plantago afra*, *Plantago ovata*, *Linum usitatissimum*, and *Plantago indica*^[85]^ and release of anthraglycosides as in *Rhamnus frangula*, *Aloe ferox*, and *Rhamnus purshiana*.^[86]^ Seed oil contains large amount of fatty acids as in *Linum usitatissimum* and can induced laxative effect by increasing contractility of the intestinal muscle.^[87]^ Recently, Laxative effect and increase water content of the colonic lumen can be induced through increasing butyric acid levels as well as by the release of acetylcholine and histamine as in *Rheum palmatum*.^[88]^

Many controlled trials indicated the indispensable benefits and promising efficacy of CHMs in nonoperative management of adhesive small bowel obstruction.^[89–91]^ The key outcomes reported in most of these trials were effects on abdominal discomfort, constipation defection, abdominal distension and reoperation rate and time of first defecation following surgery. However, the mechanism of action were not systematically described and because none of the reviewed trials discussed adverse events and safety, the CHMs effect still cannot meet the expectations and needs of patients and clinicians.

Previous review involving 1822 patients did not provide strong evidence concerning the use of CHMs in stopping bleeding from hemorrhoids.^[92]^ They postulated that, in the short term, some herbal formulae may alleviate some symptoms caused by hemorrhoids including bleeding, congestion and inflammation of perianal mucosa. Recently, data from clinical trials and systematic reviews demonstrated efficacy of CHMs in the treatment of hemorrhoids.^[93–95]^ They reported significant improvement, mainly in term of stopping bleeding, decrease in inflammatory activity, anal irritation and in the clinical and endoscopic patterns of the disease. Nevertheless, several side effects and complications were found, and the reviews displayed poor quality.

Evidence from meta-analyses support the potential benefit of CHMs when added to bio-medicine in management of dysfunctional uterine bleeding.^[59,96,97]^ The mechanism may include improvement of blood supply, estrogens-like effects and anti-inflammatory effects. Experiments in animals have demonstrated that Chinese herbal formulae containing Radix Astragali, Radix Angelicae Sinensis, and Folium Epimedii can ameliorate menopausal symptoms via an activity on estrogen regulation and can restore the blood supply and number of follicles in the ovaries, thicken the uterine wall and increase the endometrium gland.^[96,98]^

Herbal medicine have been used with potential benefits in conjunction to CM in management of postoperative acute respiratory infections^[99,100]^ and in fungal infections.^[101,102]^ However, the quality of these studies was generally poor when evaluated for data analysis, outcome measurement and treatment description. Medicinal plants including *Aloe vera*, *Centella asiatica*, *Arctium lappa*, *Curcuma longa*, *Carthamus tinctorius*, and *Paeonia suffruticosa* possess a robust and effective repair mechanism and considered as popular wound healing products.^[103–106]^ Evidence has demonstrated that these medicinal plants have a wide range of antimicrobial activity,^[107–109]^ anti-inflammatory,^[67,68]^ and antioxidant properties.^[110,111]^ The root of *Rehmannia glutinosa* has been used effectively in the treatment of diabetic foot ulcers.^[112]^

#### 3.2.2. Psychological and physical approaches

Complementary therapies that integrate physical and psychological components encompass practices such as acupuncture, massage, tai chi, yoga, spinal manipulation, and expressive therapies like art, music, and dance. Approaches like mindfulness-based stress reduction also fall within this category, along with various other holistic techniques aimed at enhancing overall well-being.^[113]^ A review of the medical literature demonstrated that these techniques can provide relaxation and reverse the negative health effects of chronic stress and improve both mental and physical health by decreasing levels of stress hormones in the body.^[114–116]^

##### 3.2.2.1. Acupuncture

Acupuncture involves the insertion of fine needles into specific points on the body to promote healing and alleviate pain.^[117]^ In surgical care, systematic reviews of RCTs demonstrated efficacy and absence of serious adverse effects of acupuncture in perioperative analgesia.^[53,118–120]^ Recently, acupuncture techniques in form of transcutaneous electric acupoint stimulation, electro acupuncture, sterile press needles acupuncture, and traditional filiform needle acupuncture appear to be most promising as adjuncts to multimodal perioperative analgesia.^[121,122]^ Studies reported that patients who received acupuncture had less postoperative pain, decrease opioid requirement, and reduce unwanted side effects of anesthesia, surgery, and opioid administration such as nausea and vomiting.^[121,123]^ In addition, acupuncture is proved to be effective in reduction of nausea and vomiting in surgical setting under combined spinal-epidural anesthesia via P6 stimulation.^[124–126]^ Acupuncture, as a complementary therapy to topical pharyngeal anesthesia, has also been indicated to be effective in relieving discomfort during gastrointestinal endoscopy.^[127,128]^ Accumulated evidence suggest that acupuncture may enhance the immune system, potentially reducing the risk of postoperative infections.^[129]^ Acupuncture has demonstrated potential benefits and promising results on tonsillectomy pain,^[130]^ after neck surgeries^[131]^ and nasal septoplasty.^[132]^ Even though, the benefits of acupuncture in surgical care has been highlighted in numerous studies, it is vital to note that the consistency and quality of evidence vary. Large-scale, high-quality clinical trials are needed to further substantiate these findings and integrate acupuncture more widely into conventional surgical care practices.

##### 3.2.2.2. Music therapy

Music therapy is a therapeutic practice that uses music to address physical, emotional, cognitive, and social needs of individuals.^[133]^ Numerous studies and clinical trials have highlighted the positive impact of music therapy in surgical care. For example, a meta-analysis of 92 randomized clinical trials examining music therapy for pain and anxiety in surgical care found that music significantly reduced pain and anxiety in surgical patients.^[134]^ Furthermore, studies have demonstrated the efficacy of music therapy in reducing negative emotions, relieving pain, and even stabilizing physiological states.^[135,136]^ Other studies have shown that music therapy can lead to shorter hospital stays and higher patient satisfaction.^[137–139]^ Music therapy is likely to become an increasingly standard component of comprehensive surgical care. Ensuring that the music chosen aligns with the patient’s preferences is crucial, as not all music has the same effect on every individual.

##### 3.2.2.3. Reflexology

Reflexology, involving the application of pressure to specific points on the feet, hands, and ears, has gained attention in various medical fields, including surgical care. A RCT of 156 patients following abdominal surgery, emphasized its effectiveness in increasing patient satisfaction, normalizing vital signs and surgical pain management, possibly through the release of endorphins and other natural pain-relieving chemicals in the body.^[140]^ In a recent review, reflexology has been implemented and considered effective in reducing postoperative pain and anxiety following total abdominal hysterectomy.^[141]^ Reflexology, as an adjunct to conventional medical treatments, has been found to be effective in reducing postoperative nausea and vomiting, a common side effect of anesthesia and surgical procedures.^[142]^ While there is growing interest in reflexology, it is important to note that scientific evidence supporting its effectiveness is still evolving.^[143]^ Some studies have demonstrated beneficial outcomes,^[141,142,144–146]^ while others suggest that more rigorous research is needed to confirm its benefits conclusively.^[147–149]^

##### 3.2.2.4. Tai Chi

Application of Tai Chi, a meditative practice, as an adjunct to physical therapy offers a range of potential benefits in surgical care, from reducing anxiety and pain to improving physical function and recovery.^[150]^ Integrating Tai Chi into preoperative routines can help patients build mental and physical resilience, improving overall fitness, improving blood flow and reducing anxiety before surgery.^[151,152]^ Tai Chi can be part of postoperative care and rehabilitation program to aid in recovery. Its low-impact nature makes it suitable for patients of all ages and fitness levels, helping to restore mobility and strength gradually.^[150]^ However, it should be integrated carefully, ensuring the practice of Tai Chi under the guidance of a qualified instructor, to ensure correct techniques and avoid injury.

##### 3.2.2.5. Yoga

Research on the potential benefits of yoga in surgical care is growing.^[153,154]^ Studies have shown that yoga, through relaxation techniques and gentle stretching, can reduce pain, lower anxiety, reduce the need for pain medications, enhance lung capacity and improve respiratory function, and improve physical function and quality of life for surgical patients.^[155–159]^ However, more extensive and rigorous research is needed to support these findings.

##### 3.2.2.6. Homeopathy

Application of homeopathy in surgical care is highly controversial and lacks robust scientific evidence.^[160,161]^ Certain remedies such as, Arnica Montana, can help manage postoperative pain, reduce anxiety and stress before surgery, reduce the need for conventional pain medications, reduce inflammation, and promote healing.^[162,163]^ Arnica Montana and Bellis Perennis were shown to be effective in reducing postoperative blood loss, seroma formation, and the need for opioids after mastectomy.^[164,165]^ However, homeopathic remedies should be used with caution and always in consultation with healthcare providers, ensuring that they do not replace or interfere with conventional medical treatments essential for safe and effective surgical care.

##### 3.2.2.7. Aromatherapy

Aromatherapy, a holistic healing technique, offers several potential benefits in surgical care, including preoperative anxiety reduction,^[166]^ pain management,^[167,168]^ and relief from nausea and vomiting.^[169]^ While the evidence supporting its use is promising, more high-quality research is needed to fully establish its efficacy and safety.

##### 3.2.2.8. Strengths and limitations of the review

This narrative review employed a broad search across multiple databases, ensuring that a wide range of relevant studies were identified. The diversity of study designs allows for a more robust synthesis of evidence, accommodating different research methodologies. This review recognizes the need for a multidisciplinary approach to integrating CIM into surgical care, involving collaboration between surgeons, anesthesiologists, and CIM practitioners to enhance the safety and effectiveness of implementation of CIM therapies. However, the heterogeneity and a wide variety of CIM practices makes it challenging to draw generalized conclusions about the efficacy of CIM in surgical care, as the effectiveness of one CIM therapy may not be applicable to another. Despite rigorous quality assessment, the review may include studies with inadequate reporting, low methodological quality, and the prevalence of various biases in the reviewed studies, potentially affecting the reliability of the review’s conclusions. Furthermore, the lack of standardized protocols for administering CIM therapies in surgical settings makes it difficult to establish clear guidelines for integrating CIM into surgical care. This narrative review highlighted the potential benefits of CIM in surgical care, however, large-scale, high-quality trials are needed to evaluate the safety and efficacy of CIM practices in surgery.

## 4. Safety considerations and conclusion

CIM offers a range of benefits that can enhance surgical care, particularly in pain management, anxiety reduction, and recovery. While promising, the integration of CIM into surgical care should be done carefully, with a strong emphasis on evidence-based practices, side effects and drug interaction consideration and collaboration with conventional medical professionals. Acupuncture presents a promising complementary approach in surgical care, offering potential benefits in pain management, anxiety reduction, and overall recovery. However, further research and careful integration with conventional medical practices are needed to maximize its potential.

Effective CIM integration requires collaboration between CIM practitioners and conventional healthcare providers. Individual assessments are necessary to determine the suitability of CIM therapies for each patient. Ensuring CIM practitioners are well-trained and certified is crucial for patient safety.

## Acknowledgments

We would like to thanks all people in the research Centre, Jazan University for their support and provision of an access to the Database. The author is gratefully acknowledge the funding of the Deanship of Graduate Studies and Scientific Research, Jazan University, Saudi Arabia, through Project Number: GSSRD-24.

## Author contributions

**Conceptualization:** Nasser Hakami.

**Data curation:** Nasser Hakami.

**Formal analysis:** Nasser Hakami.

**Funding acquisition:** Nasser Hakami.

**Investigation:** Nasser Hakami.

**Methodology:** Nasser Hakami.

**Project administration:** Nasser Hakami.

**Resources:** Nasser Hakami.

**Software:** Nasser Hakami.

**Supervision:** Nasser Hakami.

**Validation:** Nasser Hakami.

**Visualization:** Nasser Hakami.

**Writing – original draft:** Nasser Hakami.

**Writing – review & editing:** Nasser Hakami.
